# Histological and Immunohistochemical Evaluation of Autologous Cultured Bone Marrow Mesenchymal Stem Cells and Bone Marrow Mononucleated Cells in Collagenase-Induced Tendinitis of Equine Superficial Digital Flexor Tendon

**DOI:** 10.4061/2010/250978

**Published:** 2010-03-22

**Authors:** Antonio Crovace, Luca Lacitignola, Giacomo Rossi, Edda Francioso

**Affiliations:** ^1^Dipartimento dell'Emergenza e dei Trapianti di Organi (D.E.T.O.), Sezione di Chirurgia Veterinaria, Facoltà di Medicina Veterinaria, Università degli Studi di Bari, s.p. per Casamassima Km 3, Valenzano, 70010 Bari, Italy; ^2^Dipartimento di Scienze Veterinarie, Facoltà di Medicina Veterinaria, Università degli Studi di Camerino, via Circonvallazione 93-95, 62024 Matelica (MC), Italy

## Abstract

The aim of this study was to compare treatment with cultured bone marrow stromal cells (cBMSCs), bone marrow Mononucleated Cells (BMMNCs), and placebo to repair collagenase-induced tendinitis in horses. In six adult Standardbred horses, 4000 IU of collagenase were injected in the superficial digital flexor tendon (SDFT). Three weeks after collagenase treatment, an average of either 5.5 × 10^6^ cBMSCs or 1.2 × 10^8^ BMMNCs, fibrin glue, and saline solution was injected intralesionally in random order. In cBMSC- and BMMNCS-treated tendons, a high expression of cartilage oligomeric matrix protein (COMP) and type I collagen, but low levels of type III collagen were revealed by immunohistochemistry, with a normal longitudinally oriented fiber pattern. Placebo-treated tendons expressed very low quantities of COMP and type I collagen but large numbers of randomly oriented type III collagen fibers. Both cBMSC and BMMNCS grafts resulted in a qualitatively similar heling improvement of tendon extracellular matrix, in terms of the type I/III collagen ratio, fiber orientation, and COMP expression.

## 1. Introduction

The use of autologous mesenchymal cells from BM to repair experimental injuries of tendons and ligaments has been amply described in experimental animals [1, 2]. In 2003, Smith et al. [3] were the first to suggest the use of autologous BM mesenchymal cells to treat spontaneous tendon lesions in horses [4–6]. Despite these very promising results, only clinical and ultrasonographic examinations have been evaluated. 

The delivery of Bone marrow mononucleated cells (BMMNCs) has been described as an alternative procedure to grafting MSCs in injured tissues. Their successful use has been reported in the treatment of myocardial infarct lesions, bone defect reconstruction, and ischemia-reperfusion muscle injury [7–13]. The idea of using BMMNCS autografts is based on the assumption that among the mononucleated cells MSCs, which are present in only relatively small numbers in BM aspirates, can be easily separated ex-vivo (i.e., in the laboratory) from the rest of the harvested cells, concentrated in small volumes, and then immediately implanted into the patient's injured tissue. 

The aim of this study was to compare tendon healing following transplantation of cultured bone marrow stromal cells (cBMSCs), bone marrow mononucleated cells (BMMNCS), and placebo in an equine model of collagenase-induced tendinitis.

## 2. Materials and Methods

### 2.1. Horses

The study was performed at the School of Veterinary Medicine, University of Bari, after having obtained approval from the Ministery of Health, Rome, Italy (approval 4/2005 and executed under Animal care guidelines). Six stallions Standardbred horses, aged 4 years and with a mean weight of 522 ± 30 kg, not suffering from previous tendon injury, were used in this investigation following 4 weeks of stall rest prior to beginning the study.

### 2.2. Time Course Description

**Figure 1:**
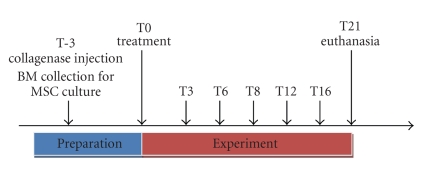
Time course description of the study performed over a period of 24 weeks (T-3-T21); US data were recorded immediately prior to collagenase injection to induce tendon injury, recorded as T-3, then 3 weeks later, just prior to injection of either cBMSC or BMMNCS suspension, fibrin glue, or placebo (= saline) into the tendon lesion (T0), and again at 3, 6, 8, 12, 16, 18, and 21 weeks (T3-T21) following tendon treatment. At T21, animals were euthanized and the SDFTs of all four limbs were harvested and processed for histology and immunohistochemistry examination.

The time course of the experiments is depicted in Figure 1. 

### 2.3. Collagenase-Induced Tendinitis Model

Four horses received collagenase in all legs, while the other two only in three legs (left fore, right fore, and right hind—the left hind limb was not injected and served as a sham control). 4000 IU of Clostridium histolyticum Type 1A collagenase (Sigma, Milan, Italy) were injected under ultrasonographic guidance into zone 2B (14 cm distal to the accessory carpal bone; 21 cm distal to the tuber calcanei) of each superficial digital flexor tendon (SDFT). Buprenorphine (6 *μ*g/kg intravenously [IV]) was administered as analgesic twice a day for 7 days. After 3 weeks, the lesions were clinically and ultrasonographically evaluated. After 21 weeks, the animals were euthanized and the SDFTs of all four limbs were harvested and processed for histology and immunohistochemistry examinations. 

### 2.4. Bone Marrow Collection

For BM aspiration, the horses were sedated with detomidine (20 *μ*g/kg IV) and butorphanol (10 *μ*g/kg IV) and restrained in a standing stock. BM was harvested on two occasions from the horses' tuber coxae, using an 8 G Jamshidi needle and heparinized 50 mL-syringes. An average of 35 mL was collected on the same day of collagenase injection (T-3) for cBMSC cultures and an average 46 mL on day 21 (T0) for processing BMMNCS, harvested from the tuber coxae on the opposite site. Buprenorphine was administered (2 *μ*g/kg) but no other analgesics or antiinflammatory drugs. The BM aspirate was immediately transported to the on-site laboratory. 

### 2.5. BMMNCS

The BM was diluted 1 : 1 in PBS, then stratified 1 : 1 on Biocoll Separating solution (FICOLL, gradient 1.077 g/mL, Biochrom, Leonopenstr 2–6, D-12247, and Berlin, Germany) and centrifuged at 2000 rpm × 30 minutes. The separated cells were counted using nuclear staining (0.1% methyl violet in 0.1 M citric acid). The cells were rinsed twice with phosphate buffer solution (PBS) and then suspended in an adequate amount of fibrin glue (Tissucol, Baxter Spa, Rome, Italy) depending on the extent of the SDFT lesion (calculated ultrasonographically) where they were to be injected. To evaluate the capacity of the cells to form colony-forming unit fibroblasts (CFU-f), 100 *μ*L of cells were seeded before and after density gradient centrifugation in two Petri dishes measuring 100 mm in diameter in complete medium (Coon's F 12 medium, 10% FBS, 100 IU/mL penicillin and streptomycin, 5% of a 200 *μ*M solution of L-glutamine (Biochrom, Leonopenstr 2–6, D-12247, Berlin, Germany)), and incubated at 37°C in a humid 5% CO_2_/air (carbogen) atmosphere for two weeks, replacing the medium twice a week. The cells were then washed with PBS, pH 7.2, fixed with buffered formalin 4% and stained with 1% methylene blue in borate buffer (10 mM, pH 8.8).

Approximately 2 h after BM collection, density gradient centrifugation of native BM aspirates yielded 122.3 ± 23.1 × 10^6^ BMMNCs that were suspended in a mean volume of 2.0 ± 0.5 mL of fibrin glue for subsequent injection into tendon lesions. 

### 2.6. Culture of BMSCs

To obtain cBMSCs from harvested BM, the mononuclear bone marrow cells were isolated by density gradient centrifugation as described above for BMMNCS and were seeded in flasks at a concentration of 4-5 × 10^6^ cells/cm^2^ in complete medium (Coon's, Biochrom, Leonopenstr 2–6, D-12247, Berlin, Germany) at 37°C in a humid 5% CO_2_/air (carbogen) atmosphere. The medium was replaced twice a week until the cells became confluent. Cells were harvested from dishes at the first passage, that is, after 3 weeks of cultivation. The BMSCs cultures were trypsin-treated in a routine manner and, after collection in Falcon tubes, the detached cells were rinsed in PBS and centrifuged twice at 1200 rpm for 10 minutes. A subsequent cell count revealed an average 5.5 × 10^6^ cells, which were suspended in a mean of 4.2 ± 2.3 mL of fibrin glue for transplantation into the tendon. 

### 2.7. cBMSC, BMMNCS, and Placebo Injection

On the day of cell transplantation (T0, 3 weeks after collagenase injection), horses were sedated as described before for BM collection and under local skin block with 1 mL lidocaine 2%, a 23 G needle was inserted laterally into the tendon lesion using transverse US scans for guidance. Volume of fibrin, cells suspension, or saline had been considered on the basis of approximately lesion size calculated by US evaluation of CSA-l (mm^2^) × length of the lesion (mm). Subsequently, either cBMSC or BMMNCS suspension, fibrin glue or saline was randomly assigned to each leg and injected by US-guidance to fill the defect in the tendon. Immediately after injection into the lesion, a small amount of thrombin (0.5 mL, 500 IU/mL; Baxter Bioscience, Pisa, Italy) was added to clot the suspension.

### 2.8. Ultrasound Examination

Ultrasound examinations were performed with GE LS-400 equipped with an 11 MHz linear probe prior to collagenase injection (T-3) and at 0 (the day of cell graft treatment), 3, 6, 8, 12, 16, 18, and 21 weeks (T0-T21) following tendon treatment. The following parameters were determined to quantify the degree and time-related changes of the SDFT lesion: tendon echogenicity score (TES), fiber pattern score (FPS), and the cross-sectional area percentage of the lesion (% CSA-l) assessed in the zone of maximum injury (MIZ), as well as the length of tendon lesion (in mm) measured in longitudinal US images. For semiquantitative TES evaluation, scores were assigned as 0 (isoechoic), 1 (hypoechoic), 2 (extremely), and 3 (anechoic) (Genovese and Rantanen [14]). The FPS describes the alignment of collagen fibers in longitudinal scans, with low values indicating a normal longitudinal fiber pattern and higher scores describing a greater degree of nonlongitudinal fiber pattern, that is, disrupted pattern: 0 (>75%), 1 (50%–74%), 2 (25%–49%), 3 (<25%) (Genovese and Rantanen, 1998). The % CSA-l refers to the ratio between the lesion cross section and the entire cross- sectional area of the SDFT.

### 2.9. Histology

Histological and immunohistochemical evaluation has been performed in blinded fashion by a single operator. Starting from the maximum injury zone MIZ, SDFTs were divided into 1 cm sections on either side of the maximum injury zone, obtaining a total of 8 blocks from each harvested tendon. Each block was fixed in 10% neutral buffered formalin, and, after 48 h, sections of 1.5 mm thickness in the longitudinal direction (frontal plane) were placed in a transilluminator for gross morphological evaluation (i.e., areas of maximum injury and disorganization of peritendon structures) and digital photographs were recorded; then tendons were furthermore sectioned according to the longitudinal axis, following the orientation of the collagen fibers arrangement and sections were paraffin embedded. Three *μ*m thick sections were obtained and stained with hematoxylin-eosin (HE) stain for histopathological examination and with Herovyci's polychrome stain for precollagen and collagen differentiation (Busoni et al. [15]). Histological examination of SDFT portions included assessment of areas of alignment of collagen fibers in a longitudinal pattern, using a fiber orientation score (FOS). Unlike the US examinations (FPS score), high FOS values indicate a normal longitudinal fiber pattern, while lower scores describe a greater degree of nonlongitudinal or disrupted pattern: 0 (>75%), 1 (50%–75%), 2 (25%–49%), 3 (<25%), where the percentages refer to the ratio of damaged area to the entire cross-sectional area of the SDFT, as assessed at a magnification of 10×. For each considered parameter (i.e., predominant percentage of aligned or unaligned-cross or fingerprint pattern organized fibers), five randomly selected fields were observed, and the score was evaluated as the mean percentage of the examined fields.

The number of mononuclear inflammatory cells such as lymphocytes, plasma cells, and macrophages was assessed at 40× magnification (high-power field—HPF). For this evaluation, ten randomly selected fields were examined per each tendon sample. The number of these cells was recorded and results were reported as the mean value for the entire specimen, and then the degree of mononucleated inflammatory cell infiltration (MICI) was scored. Number of inflammatory mononuclear cells was considered to be normal when none or only a few cells were seen in each HPF among collagen fibers (score 0). Increase in number of cells was considered mild for specimens with several cells per HPF (score 1), moderate (score 2) for specimens with many cells for HPF, or marked (score 3) for specimens with numerous to very numerous cells per HPF, respectively. Histological criteria for a normal tendon structure included detection of a perfect alignment in a parallel manner of collagen fibers, and the absence of inflammatory cells between fibers and a homogeneous character of mature collagen, revealed by intense red staining of fibers using the Herovyci method. 

### 2.10. Immunohistochemistry

To evaluate the type of collagen synthesized in damaged and normal areas and to immunohistochemically characterize the cell populations infiltrating the tendons, the following polyclonal antibodies (pAbs) were used in serial sections: rabbit pAb anti-collagen type I (Chemicon, Temecula, CA), rabbit pAb anti-collagen type III (Chemicon, Temecula, CA), rabbit pAb anti-cartilage oligomeric matrix protein (COMP; Abcam, Cambridge, UK), mouse mAb anti-vimentin (Dako, Glostrup, Denmark), and mouse mAb anti-CD34 (Clone BL-35C, Zymed Inc., San Francisco, CA). Specific primary antibodies substituted with TBS or nonimmune sera were used as negative controls in immunohistochemical techniques.

For immunohistochemistry tests, sections were placed on pretreated slides (Bio-Optica, Milan, Italy) to promote adhesion and dried overnight at 37°C. After dewaxing, sections were placed in EDTA buffer, pH 9.0, and processed in a microwave oven at 650 W for two cycles of 10 minutes each to enhance their antigenicity. Slides were then allowed to cool at room temperature for at least 20 minutes before further processing for immunostaining employing standard procedures. Tissue sections were incubated overnight in a moist chamber at 4°C with different primary antibodies, diluted 1 : 50 in Tris-buffered solution (TBS) containing 0.1% crystalline bovine serum albumin (BSA). Binding of the antibodies was detected with ABC-peroxidase (Vector Laboratories Inc., Burlingame, CA) techniques using 1 : 200 diluted biotin conjugated goat anti-rabbit immunoglobulin G (Vector Laboratories Incorporated, Burlingame, CA) and a 1 : 200 diluted biotinylated goat anti-mouse immunoglobulin (AO433; DAKO, Glostrup, Denmark), applied for 45 minutes at room temperature as secondary antibodies. The enzymatic reaction was developed with 3, 1-diaminobenzydine (DAB) (Sigma, St. Louis, MO) or VIP (Vector), as substrate for the ABC-peroxidase technique, using Meyer hematoxylin as nuclear counterstain.

Immunohistochemical evaluation was made of collagen type I or III prevalent expression in damaged and normal areas of tendon tissue, of cartilage oligomeric matrix protein (COMP) expression, and the presence of CD34 positive staining (CD34+) mononuclear cells interspersed or localized in perivascular fashion throughout the damaged area of the SDFT, assessed at 100×. A score of 0 (absence of antigen expression), 1 (weak and spotted antigen expression), 2 (weak but diffuse antigen expression throughout the entire specimen), and 3 (diffuse and strong antigen expression) was assigned to the semiquantitative evaluation of the immunohistochemical reaction to each antigen employed.

### 2.11. Statistical Analysis

Numerical data are given as mean ± SD. Two-way ANOVA (repeated measure analysis) analysis was conducted to test the treatment by time interactions, and the effect of treatment over time. If an interaction of time by time was detected at the 0.05 level of significance, then a subgroup analysis was made of the effect of treatment at each time point at the 0.05 level, using a least square difference (LSD) test as post-hoc test. 

Evaluation of histological and immunohistochemical scores was conducted by *U*-Test (Mann-Whitney) to compare treatments (significance *P* ≤ .05).

## 3. Results

### 3.1. Colony Forming Units of Fibroblasts (CFU-f) Count

The number of culture dish-adherent BM stem cells that can form a colony was quantified as CFU-f. Prior to Ficoll gradient centrifugation, the original BM aspirate in all 6 horses consisted of a mean of 428 ± 223 CFU-f per mL (pre-Ficoll) while postcentrifugation (post-Ficoll) the number rose to 1697 ± 785 CFU-f per mL. 

### 3.2. Ultrasonographic Findings

#### 3.2.1. Tendon Structure before and after Collagenase-Induced Tissue Injury

Ultrasound examination at the beginning of the study (T-3) was made to check for an intact tendon structure of the SDFT in all 4 limbs, with no evidence of previous injury. Three weeks after collagenase injection, (T0) US examination confirmed significant SDFT lesions, which were not uniform in shape and length. Nevertheless, TES and FPS scores, % CSA-l, and length of lesion at T0 were not different among limbs or horses (*P* > .05). The experimentally induced lesions in all 22 SDFTs were on average 38.8 ± 10.4 mm long, with a lesion CSA to tendon CSA ratio of 30.1 ± 11.6% at the MIZ. At T0, TES, and FPS scores were 2.9 ± 0.3 and 2.9 ± 0.3, respectively.

#### 3.2.2. Tendon Structure following Treatment

Between T3 and T6, SDFT lesions were still well appreciable in US images although a progressive improvement in the TES and FPS scores as well as the % CSA-l was noted, but without any significant difference among differently treated tendons. 

**Figure 2:**
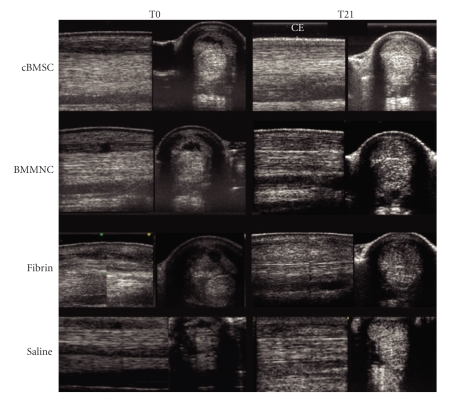
Longitudinal and transverse ultrasound images recorded on T0, that is, the day of injection of intralesional cultured bone marrow mesenchymal cells (cBMSC), bone marrow Mononucleated Cells (BMMNCS), fibrin and saline, and at T21, 21 weeks after treatment.

At T8, US determination of % CSA-l revealed no statistical difference between tendons treated with cBMSCs or BMMNCS, whereas the % CSA-1 was significantly larger in saline and fibrin-injected SDFTs (*P* < .05). At this time point, TES and FPS scores were still different from baseline (T-3) values, but had significantly improved as compared to the examination at T0 (*P* < .05). From T16 on up to T21, the TES, FPS, and % CSA-l values in cBMSC- and BMMNCS-injected SDFTs were significantly different from those measured in fibrin and placebo-treated SDFTs (*P* < .05). US scans are depicted in Figure 2.

### 3.3. Histology Findings

**Figure 3:**
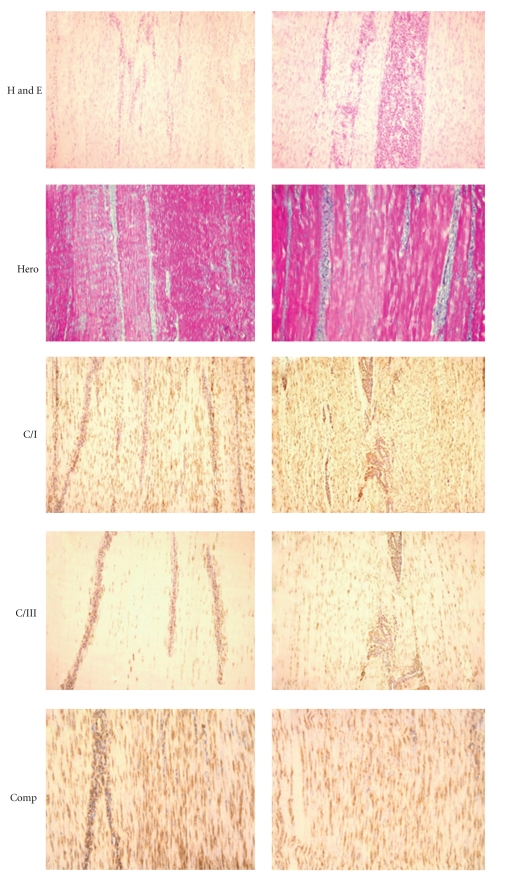

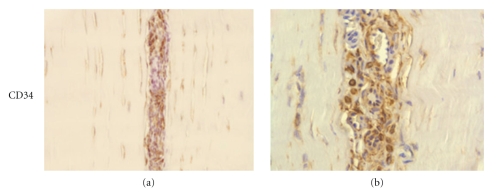
Tissue slides obtained from SDFTs injected with cBMSCs, and BMMNCS. Tissue stains with hematoxylin-eosin (H and E) and Herovyci (Hero) highlight the longitudinal orientation of collagen fibers after transplantation of cBMSCs and BMMNCS; Histological examination of the sham tendons, both with HE and Herovyci staining, showed the presence of mature collagen (i.e., predominantly strong red Herovyci staining of fibers in tendon sections) and a longitudinal fiber orientation, signifying a normal tendon architecture. Additionally, the absence or very scanty mononuclear cell infiltrate was observed in all normal control tendons. No inflammatory or other reactions to the fibrin glue were observed in any of the treated limbs, and at T21 no fibrin residues were recognized in microscopic examinations. (T21) Tissue slides from the superficial digital flexor tendons (SDFT) following experimentally induced tissue injury and subsequent treatment with cultured bone marrow mesenchymal stem cells (cBMSCs (a)) and Bone Marrow Mononucleated Cells (BMMNCS (b)). Incomplete maturation of collagen was verified by the observation of blue staining fibers mixed with red ones. In BMMNCS a large number of mononucleated cells are recognizable in the interfascicular zone. Immunohistochemistry stains for Collagen type I (C/I) and collagen type III (C/III) show a high expression of C/I and a very low expression of C/III. Stains for cartilage oligomeric matrix protein (COMP) expression. BMMNCS-treated SDFT (b) show a strong, diffuse expression like sham SDFT (Figure 4(c)). Immunohistochemistry staining for the expression of CD34+ mononucleated cells in cBMSCs (a) shows a low expression limited to the interfascicular zone. In BMMNCS (b)-treated SDFT, the presence of numerous mononucleated CD34+ cells near microvessels, which sometimes show some CD34+ endotheliocytes in their wall, which is highlighted. In cBMSC- and BMMNCS-treated SDFTs, microscopic examination at T21 revealed a longitudinal orientation of newly formed collagen fibers. At this time point, sections stained by the Herovyci method revealed minimal areas of blue-stained thin, well-oriented precollagen fibers mixed with well-differentiated and- oriented red-stained mature collagen fibers, indicating a still somewhat incomplete collagen maturation at this time in the treatment. In BMMNCS-treated SDFTs also, large numbers of mononucleated cells were present in the interfascicular zone. In contrast, in placebo SDFTs, injected with saline or fibrin, the tissue architecture was disrupted or markedly modified, showing blue precollagen and red collagen fibers randomly or not longitudinally oriented, and a relatively loss of the architectural pattern.

**Figure 4:**
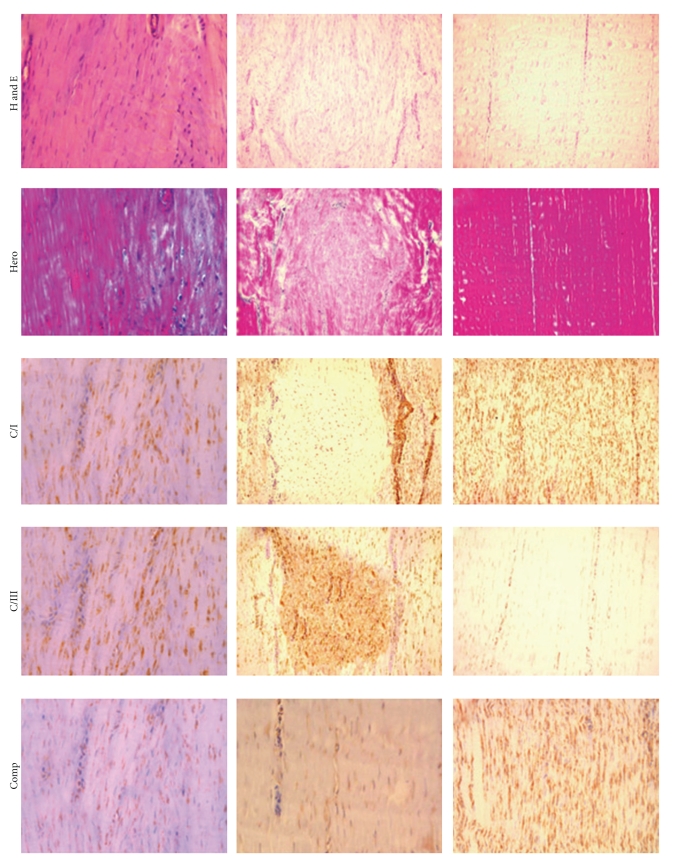

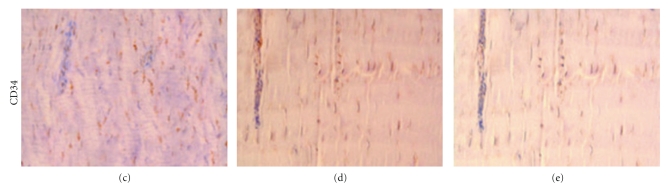
(T21) Tissue slides from the superficial digital flexor tendons (SDFT) following experimentally induced tissue injury and subsequent treatment with Fibrin (c), Saline (d), and normal tendon (e). Tissue stains with hematoxylin-eosin (H and E) and Herovyci (Hero) show loss of the longitudinal fiber pattern and crimp in tendons treated with-Fibrin (c); in Saline treated tendon (d), there is complete disruption with randomly oriented fibers, clearly shown in the central area. The collagen I/III ratio in Fibrin treated tendon (c) is clearly lower than in normal (e) and cell grafted tendon (Figures 3(a) and 3(b)); in Saline (d) treated tendon, the central zone was highly positive for collagen type III but not for type I. Stains for cartilage oligomeric matrix protein (COMP) show stack and spread expression in both control-treated SDFT (c-d). In immunohistochemistry staining for the expression of CD34+ mononucleated cells in Fibrin (c), Saline (d) and Normal (e) Tendon, only sporadic CD34+ stained mononuclear elements were detected. By contrast, in all three placebo-treated SDFTs, the location of the lesion was readily identified as an area with high positivity for type III collagen, very low expression of type I collagen and in some zones, evidence of mineralization. Similarly to the trend of type I collagen expression, COMP was expressed in a homogeneous and diffuse pattern in sham untreated control SDFTs as well as in cBMSC- and BMMNCS-injected tendons. Scant, weak expression of COMP, restricted to areas that circumscribed the injected portions of the tendon, was observed in all three placebo-treated SDFTs. As expected, no CD34+ mononuclear cells were observed in the sham tendon sections. Similar results were obtained in sections of placebo-injected SDFTs, although a certain number of inflammatory cells were present, interspersed in perivascular areas of these tendons (data not shown). Only sporadic CD34+ stained mononuclear elements (i.e., 0 to 2 per HPF) were observed in cBMSC-injected tendons (data not shown). On the contrary, in BMMNCS-treated SDFTs large numbers of mononucleated cells, in part CD34+ stained, were present in the interfascicular zone.

 Representative histological slides in the experimental horses are shown in Figures 3 and 4. 

Scores describing the changes in morphological (fiber orientation and mononuclear cells infiltration in SDFTs) and immunohistochemical parameters (degree and intensity of collagen I/III, COMP, and CD34 expression) are summarized in Table 1. 

### 3.4. Immunohistochemistry Findings

Immunohistochemical stains for type I and III collagen revealed a high expression of type I collagen in cBMSC- and BMMNCS-injected tendons, but very low expression of type III collagen (Figures 3 and 4). 

Scores describing the changes in immunohistochemical parameters (degree and intensity of collagen I/III, COMP and CD34 expression) are summarized in Table 1.

## 4. Discussion

The present results provide evidence that regeneration of injured tendon tissue can be achieved in the horse with intralesional injection of both cBMSCs and BMMNCS, while placebo (saline or fibrin) injection leads to scar tissue repair of the damaged site. Collagen expression was equally well restored in both cell graft groups, with type I collagen fibers largely predominating over type III collagen fibers. Interestingly, our study confirms that COMP, an abundant noncollagenous pentameric glycoprotein in the tendon [16], is well expressed in normal equine SDFTs, and the level of expression is strictly related to a high percentage of physiologically oriented collagen type I fibers. Similar results were observed in both cBMSC- and BMMNCS-injected tendons but not in placebo controls. Previous studies demonstrated the ultrastructural distribution of COMP and its correlation to collagen fiber thickness in different compartments of equine flexor tendons, with a particular concentration of this protein at the gap region of collagen type I and II fibers [17]. The structure and organization of the ECM, and in particular the axial alignment of type I collagen fibers, are essential for the tensile strength of tendons and are mainly related to the COMP presence and distribution [17].

Type I collagen fibers were longitudinally oriented in cell-graft injected tendons, indicating a physiological texture of the repaired tissue sites. Following both cBMSC and BMMNCS treatments, the tendon ECM did not differ in terms of the type I/III collagen ratio and fiber orientation from intact SDFT tissue (tendons in sham controls). By contrast, in placebo-treated lesions type III collagen formation predominated while type I collagen was only marginally expressed and the fibers were randomly oriented, indicating the loss of a normal tissue structure. As to the presence of some immature collagen in both cBMSC- and BMMNCS-treated tendons, we can only speculate that the finding could be due to minimal mechanical tendon load as a result of prolonged stall rest. 

BMMNCs are characterized by CD34+ surface marker while BMSC express the surface marker CD34+ during an early phase but lose it in adult cells [18]. Thus the presence of cells that express the marker CD34+ in treated tendons is the evidence of a chemotactic effect promoted by the MSCs. Additionally, in our opinion, the presence of a certain percentage of CD34+ mononuclear cells localized in perivascular areas of the interfascicular zone of BMMNCs-treated SDFTs pointed toward the possible angiogenic role by endothelial differentiation of these cells. Thus, the presence of cells expressing CD34+ in tendons provides evidence that cBMSCs and BMMNCS home into the tendon after treatment, and presumably they have an attractive effect for local CD34+ cells into the treated lesion. Additionally, in our opinion, the presence of a certain percentage of CD34+ mononuclear cells localized in perivascular areas of the interfascicular zone of BMMNC-treated SDFTs suggested the presence of surviving clones of injected cells and, in relation to their location, a possible angiogenic role of these cells by endothelial differentiation. 

The value of cBMSCs in the treatment of lesions in bone, skeletal and cardiac muscle, and other mesenchymal tissues has already been demonstrated [1, 2]. This also applies to equine tendon injuries and was described in a study of 235 horses treated with autologous cultured mesenchymal cell grafts [3]. However, the application of this technique requires a fairly sophisticated cell culture laboratory and highly qualified personnel, both of which are often not accessible to clinicians and/or are cost-prohibitive for veterinarians and animal owners. In addition, at least 3-4 weeks elapse between BM collection and the time of cBMSC transplantation into the tendon lesion, a delay that may have a negative impact on the quality and speed of tissue healing. It has been demonstrated that extensive in-vitro proliferation seems to affect both the replication potential of BM-derived MSCs and their differentiation capacity [19–23]. In fact, after the first confluence, the proliferation rate of cultured BM stem cells slows down; as a result of in-vitro expansion, the bone-forming activity of BM stem cells in vivo is dramatically decreased as compared to fresh BM stem cells and their in-vitro multidifferentiation potential which is gradually lost [19, 23]. This loss of in-vivo differentiation potential after in-vitro expansion drastically limits the potential use of BM-derived stem cells for therapeutic purposes [22].

Although in the present study both cBMSC and BMMNCS injections produced an equally good regeneration of tendon tissue in terms of the quality of Collagen I/III ratio expression and architecture and COMP, BMMNCS transplantation is a simpler as well as a more time- and cost-effective procedure than cBMSC therapy. The separation of BMMNCs from BM aspirate (BMMNCS) makes it possible to obtain a large number of MSCs in a short period of time (only 2 h from BM harvesting) by employing only a one-step technique (Ficoll gradient centrifugation) without the need for cell culture. The BMMNCS-based grafts can then be concentrated in the small injected volume needed to fill the tissue lesion. To transplant the same number of MSCs using native BM aspirate, a much larger volume has to be injected into the tendon, that could create an iatrogenic tendinitis of normal tendon tissue, and other possible complications such as ossification and the delivery of exogenous tissue (i.e., bone spiculae, fat, etc.). Once transplanted into the lesion site, the ability of MSCs included in BMMNCS to immediately clone and differentiate into tendon tissue, forming fibroblasts, helps to avoid the significant time delay in therapy associated with the use of cBMSCs and thus may offer the advantage of a more rapid tissue healing and a sooner return of tendon-injured horses to athletic performance level.

However, the caveat of having enrolled only a small number of horses in this initial investigation and the potential disadvantage of having studied the effects of MSC transplantation in an experimental model of tendon injury may limit the implications of our findings for clinical practice and calls for a more controlled (ideally blinded) experimental study and a subsequent clinical follow-up study in a larger number of horses. 

In conclusion, this study is the first to describe the efficacy of BMMNC injection in an equine model of experimental tendinitis. The use of BMMNCS offers significant advantages in terms of the progress and quality of tendon healing when compared to native BM implantation. Although both cBMSC and BMMNCS injections produced a comparably positive effects on the regeneration of tendon tissue particularly in terms of the collagen I/III ratio and COMP expression, the use of BMMNCS is a simpler and more cost-effective method than the use of cultured BMSCs, and thus its introduction into equine clinical practice may well have a significant impact on tendon injury treatment in the future. 

## Figures and Tables

**Table 1:** Morphological and immunohistochemical scores evaluated for each horse at 21 weeks following treatment of collagenase-induced tendinitis SDFT, superficial digital flexor tendon; RH, right hind limb; LF, left fore limb; RF, right fore limb; cBMSCs, cultured bone marrow mesenchymal cells; BMMNCS, bone marrow mononucleated cells; FOS, fiber orientation score; MCI, mononucleated cell infiltration score; Collagen I, type I collagen fiber expression score; Collagen III, type III collagen fiber expression score; COMP, cartilage oligomeric matrix protein expression score; CD34, score describing presence of CD34+ mononuclear cells localized throughout the damaged area of the SDFT (see Section 2 for more details). Significant differences from placebo group: #*P* < .05.

Treatment	Histological and immunohistochemical scores					
	FOS	MCI	Collagen I	Collagen III	COMP	CD34
cBMSCs	3.0	1.6	2.6	1.2	2.2	1.0
sd	0.0	0.5	0.5	0.4	0.4	0.7
BMMNCS	3.0	2.4	3.0	1.4	2.4	2.0
sd	0.0	0.5	0.0	0.5	0.5	0.7
Fibrin	0.5	0.5	1.0	3.0	0.5	0.0
sd	0.7	0.7	0.0	0.0	0.7	0.0
Placebo	0.8	0.5	1.3	2.3	1.3	0.0
sd	1.0	0.6	0.5	0.5	0.5	0.0
